# Using Visual Aids to Improve Communication of Risks about Health: A Review

**DOI:** 10.1100/2012/562637

**Published:** 2012-05-02

**Authors:** Rocio Garcia-Retamero, Yasmina Okan, Edward T. Cokely

**Affiliations:** ^1^Department of Experimental Psychology, University of Granada, 18071 Granada, Spain; ^2^Center for Adaptive Behavior and Cognition, Max Planck Institute for Human Development, 14195 Berlin, Germany; ^3^Department of Cognitive and Learning Sciences, Michigan Technological University, Houghton, MI 49931, USA

## Abstract

Recent research has shown that patients frequently experience difficulties understanding health-relevant numerical concepts. A prominent example is *denominator neglect*, or the tendency to pay too much attention to numerators in ratios (e.g., number of treated patients who died) with insufficient attention to denominators (e.g., overall number of treated patients). Denominator neglect can lead to inaccurate assessments of treatment risk reduction and thus can have important consequences for decisions about health. Here, we reviewed a series of studies investigating (1) different factors that can influence patients' susceptibility to denominator neglect in medical decision making—including numerical or language-related abilities; (2) the extent to which denominator neglect can be attenuated by using visual aids; and (3) a factor that moderates the effectiveness of such aids (i.e., graph literacy). The review spans probabilistic national U.S. and German samples, as well as immigrant (i.e., Polish people living in the United Kingdom) and undergraduate samples in Spain. Theoretical and prescriptive implications are discussed.

## 1. Introduction and Background

Many modern health messages seem to suggest that we live in an era of medical wonders. Health care professionals and the media alike are reporting that mammography screenings reduce the risk of dying from breast cancer by 25% [1], and prostate-specific antigen tests cut deaths from prostate cancer by 20% [2]. It seems that patients today are in good hands and can simply relax and follow their doctors' advice—medical tests and treatments catch diseases early and save lives.

Unfortunately, although medicine has advanced at an extraordinary rate within the last century, some promises like those mentioned above are still overly optimistic. While the information presented is accurate according to many experts, it is provided in a format that makes medical screenings and treatments seem more beneficial than they actually are. To illustrate, the 25% reduction in risk of dying of breast cancer means that without mammography 4 of 1,000 women will die of breast cancer, compared to 3 who die even though they participate in regular screenings [3]. Even this low estimate has been debated in recent reviews [4, 5], showing that for every 2,000 women screening will prolong the life of only 1 woman, but falsely diagnose 10 women who are in fact healthy. Similarly, the claim for 20% fewer deaths of prostate cancer due to PSA screening masks the fact that the overall mortality remains the same: An equal number of men die with and without the PSA screening, but among those who participate in screening, deaths are more often attributed to causes other than prostate cancer [6]. In sum, it comes as no surprise that although progress is steady, medicine is not an exact science. Even the best available medical procedures can be burdened with uncertainties, may be ineffective, and sometimes do more harm than good. When information about such procedures is not transparent, neither doctors nor their patients can make accurate, informed medical decisions.

Why are benefits of medical screenings and treatments so often presented in a nontransparent way? In part, the problem lies in the lack of an awareness of potential biases and alternative options. There is simply not enough awareness that the same information can be presented in different ways and lead to different conclusions. In fact, many people do not understand the relationship between the different ways in which probabilistic information can be expressed [7–11]. This is true not only among the general population, but also among medical experts who often have problems recognizing limits of information formats. Of note, problematic numerical presentations appear even in high ranking medical journals [12].

Ratio concepts—of which risks and probabilities are examples—are particularly challenging and prone to biases that undermine good judgment and decision making [13, 14]. A prominent example of people's difficulties with ratio concepts is *denominator neglect *[15–17]. That is, people often pay too much attention to the number of times a target event has happened (numerators) and insufficient attention to the overall number of opportunities for it to happen (denominators; [16]). Denominator neglect has been studied both in medical and nonmedical contexts [18–21]. To illustrate, in an experiment by Yamagishi [22], participants were presented with estimates of the number of deaths in the population due to eleven causes (e.g., cancer) and had to assess the risk of dying of such causes. These estimates were presented both as numbers of deaths out of 10,000 and of 100. Participants rated the likelihood of a cancer killing 1,286 out of 10,000 people (i.e., 12.86%) as higher than 24.14 out of 100 people (i.e., 24.14%). The degree of perceived riskiness, therefore, varied according to the number of deaths presented (numerators), irrespective of the total possible number of deaths (denominators).

**Figure 1:**
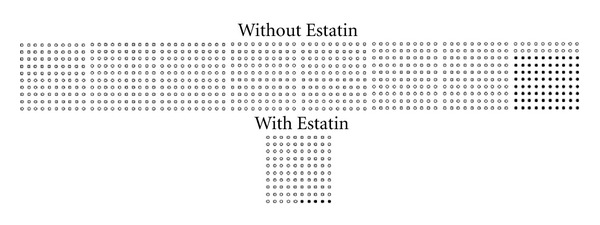
Numerical information about relative risk reduction and additional visual information (icon array). A new drug for reducing cholesterol, Estatin, decreases the risk of dying from a heart attack for people with high cholesterol. Here are the results of a study of 900 such people: 80 out of 800 of those who did not take the drug died of a heart attack, compared with 5 out of 100 of those who took the drug.

Denominator neglect can have important consequences when making decisions about health. In medical practice, for example, the overall number of patients who receive a certain treatment is often smaller than the number of those who do not [23, 24]. Therefore, patients and their doctors might be able to think of more people who did not have a particular screening or take a novel drug than those who did. If individuals disregard the overall number of treated and nontreated patients (e.g., 100 and 800, resp.), they might perceive the treatment to be more effective than it actually is. That is, they might compare the absolute numbers of treated and nontreated patients who die (e.g., 5 and 80, resp.) rather than the proportion of treated and nontreated patients who die (e.g., 5 of 100 and 80 of 800 for a treatment risk reduction of 50%; see Figure 1). Notably, most of the past research examining people's perceptions of treatment risk reduction has employed samples of treated and nontreated patients of the same size (see [7, 25]), and even experts in medical decision making recommend doing so [26–28]. As an exception, Garcia-Retamero et al. [29] conducted a study with unequal samples of (hypothetical) treated and nontreated patients and showed that participants overestimated risk reduction when the overall number of treated patients was lower than the overall number of patients who did not receive the treatment.

A number of important factors can influence people's susceptibility to denominator neglect when estimating treatment risk reduction. In this paper, we review a series of studies investigating how individual differences in numerical and language-related skills tend to affect people's expression of denominator neglect and, in turn, the accuracy of their risk understanding [29–31]. Additionally, the studies reviewed examine the effectiveness of visual aids for improving accuracy of risk understanding among individuals disadvantaged by their lower levels of numerical skills or limited language proficiency. Finally, we review a study demonstrating that individual differences in the ability to understand graphically presented information can play a key role in the effectiveness of visual aids designed to enhance risk understanding [32]. Of note, the studies reviewed here investigated the effect of denominator neglect not only in laboratory settings in Spain but also among probabilistic national samples from two countries with very different medical systems (the United States and Germany), as well as examining decision making by immigrants (i.e., Polish people living in the United Kingdom).

## 2. The Impact of Numeracy on the Assessment of Treatment Risk Reduction

 Numeracy involves knowledge of basic mathematical and statistical operations which give rise to an understanding of basic probability and numerical concepts [8, 10, 33, 34]. Numeracy is necessary for the accurate evaluation of a variety of financial, consumer, and particularly health-relevant risk communications. Low numeracy can lead to undesirable consequences such as difficulties following dosing regimens [35], higher histories of hospitalization [36], and larger susceptibility to health information framing effects [11, 37]. Moreover, people with low numeracy are less willing to participate in decision making about health [31, 38].

To what extent can individual differences in numeracy affect understanding of treatment risk reduction? This question was addressed by Garcia-Retamero and Galesic [31] (see also [39]) in a study involving probabilistic national samples in the United States and Germany, including participants with varying levels of numeracy. In particular, the authors investigated the tendency of participants who were representative of the entire U.S. and German populations to show denominator neglect when judging the effectiveness of treatments presented with unequally sized groups of treated and nontreated patients (i.e., inconsistent denominators). As noted above, in such situations denominator neglect can be particularly problematic, leading people to show inaccurate estimates of treatment risk reduction.

Garcia-Retamero and Galesic [31] further investigated the extent to which people could be aided when making decisions about their health by means of displays designed to enhance comprehension, namely, icon arrays [26, 40]. Icon arrays (i.e., graphical representations consisting of a number of circles or other icons symbolizing individuals who are affected by some risk [26, 28, 41]) have been shown to be a promising method for communicating treatment risk reduction (see [25, 42–45]). Such visual displays can help people represent the overall number of patients who did and did not receive a treatment, thus contributing to reduce denominator neglect. That is, they enable people to disentangle classes that are overlapping in ratios, making part-to-whole relations visually available and salient (e.g., [16, 46]; see also [26]).

Participants in the study (*n* = 513 in the United States and *n* = 534 in Germany) completed a numeracy test consisting of nine items selected from Schwartz et al. [10] and Lipkus et al. [8]. For the analyses, participants were split into two groups according to the median numeracy score in the scale for the total sample (i.e., 6; see Peters et al. [11] for a similar procedure). In addition, participants were presented with a medical scenario of the usefulness of “Estatin”—a hypothetical drug for reducing cholesterol that also decreases the risk of dying from a heart attack with a relative risk reduction of 50%. In one condition, for instance, participants received the following information: “A new drug for reducing cholesterol, Estatin, decreases the risk of dying from a heart attack for patients with high cholesterol. Here are the results of a study of 900 such patients: 80 out of 800 of those who did not take the drug died of a heart attack, compared with 5 out of 100 of those who took the drug.”

Two independent variables were manipulated between groups in the study. First, the overall numbers of treated and nontreated patients (i.e., the sizes of the denominators) were set to be 800/800, 100/800, 800/100, or 100/100, where the first and second quantities reflect the overall numbers of patients who did and did not take the drug, respectively. To achieve a relative risk reduction of 50%, the sizes of the numerators (i.e., the number of treated and nontreated patients who died) varied within conditions depending on the sizes of the denominators (see Table 1).

Second, half of the participants received—in addition to the numerical information about risk reduction—two icon arrays presenting the risk of dying of a heart attack when the drug was and was not taken, respectively. All icon arrays contained either 800 or 100 circles depending on the overall number of patients who did and did not take the drug. Deceased patients were shown as black circles at the end of the array. An example of the condition involving icon arrays is shown in Figure 1.

Participants' estimates of treatment risk reduction were measured as a dependent variable. First, following the procedure used by Schwartz et al. [10], participants were asked how many of 1,000 patients with high cholesterol might die of a heart attack if they did not take the drug. Second, they were asked how many of 1,000 patients with high cholesterol might die of a heart attack if they did take the drug. The relative risk reduction estimated by each participant was calculated by subtracting the answer to the second question from the answer to the first one, and dividing it by the answer to the first. Participants were then classified depending on whether their estimates were accurate, lower, or higher than the exact value (i.e., 50%). Estimates were considered to be accurate only when they were exactly correct.

**Figure 2:**
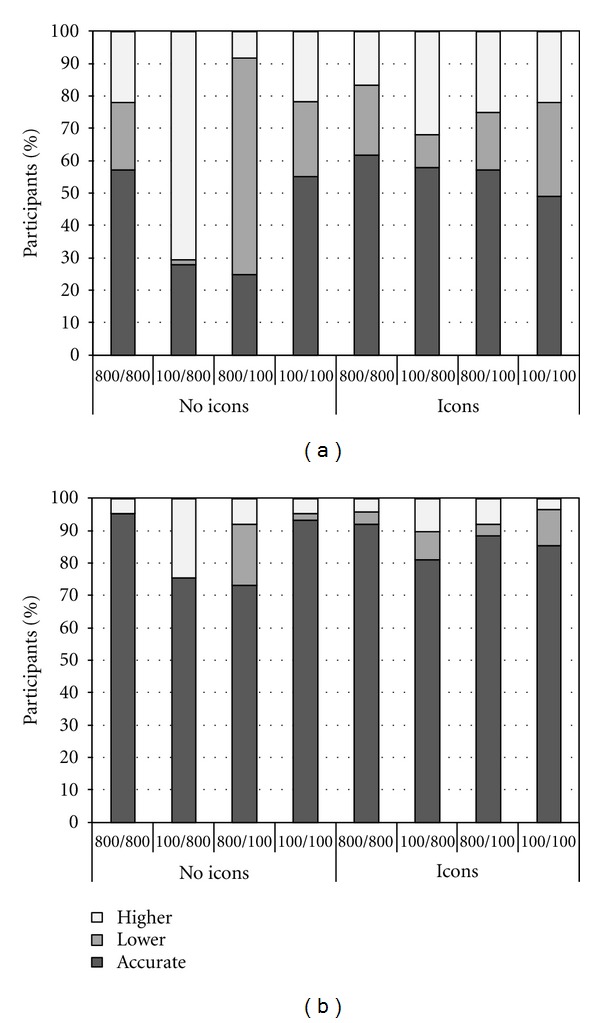
(a) Percentage of participants with *low* numeracy whose estimates of risk reduction were accurate, lower, or higher than the exact value as a function of the sizes of the denominators and icon arrays. (b) Percentage of participants with *high* numeracy whose estimates of risk reduction were accurate, lower, or higher than the exact value as a function of the sizes of the denominators and icon arrays.

Figures 2(a) and 2(b) show the percentage of participants with low and high numeracy, respectively, whose estimates of risk reduction were accurate, lower, or higher than the exact value, as a function of the sizes of denominators and icon arrays. Results showed that when information about the drug was provided numerically only (i.e., no icon arrays were presented) and the sizes of the denominators were different, many participants provided inaccurate estimates. Crucially, this tendency was larger for participants with low numeracy. In particular, when the number of treated patients was lower than the number of those who did not receive the treatment (i.e., in the 100/800 condition), 71% of the participants with low numeracy *overestimated* risk reduction, as compared to 25% of the participants with high numeracy. Note that in such a case, the number of patients who received the treatment and died (*n* = 5) is lower than the number of patients who did not receive the treatment and died (*n* = 80; see Table 1). The tendency to focus on the absolute numbers in the numerators instead of taking into account proportions (i.e., denominator neglect) can account for these findings. As a result, participants in this condition—especially those with low numeracy—frequently believed that the treatment had a *larger* effect than it actually did.

In contrast, when the number of treated patients was higher than the number of patients who did not receive treatment (i.e., in the 800/100 condition), 67% of the participants with low numeracy *underestimated* risk reduction, as compared to 19% of the participants with high numeracy. In such a case, the number of patients who received the treatment and died (*n* = 40) is higher than the number of patients who did not receive the treatment and died (*n* = 10; see Table 1). Denominator neglect can also account for these results, leading participants—especially those with low numeracy—to believe that the treatment had a *smaller* effect than it actually did. Finally, when the sizes of the denominators were equal, estimated risk reduction was inaccurate in only 56% and 6% of the participants with low and high numeracy, respectively. In these conditions, participants did not necessarily have to take proportions into account to make accurate estimates but could rely on the absolute numbers in the numerators.

Interestingly, when icon arrays were added to the numerical information, denominator neglect was significantly reduced. Notably, icon arrays were particularly helpful to reduce denominator neglect for participants who were less skilled in using numerical information. In particular, when the sizes of the denominators were different and icon arrays were added to the numerical information, the percentage of participants with low numeracy who estimated treatment risk reduction incorrectly decreased from 74% to 42% and from 26% to 15% among participants with high numeracy. Taken together, these results suggest that numeracy is a key factor that can moderate the effect of denominator neglect. Overall individuals with low numeracy are more likely to show biased and inaccurate estimates of risk reduction. Fortunately, icon arrays are particularly effective for enhancing comprehension among such individuals. Results also show the generalizability of denominator neglect and the effect of icon arrays on two different cultures.

## 3. The Impact of Language Skills on the Assessment of Treatment Risk Reduction

 The interpretation of health-related risk information not only requires advanced knowledge of statistical concepts but also language proficiency [47]. Thus, another factor that can significantly affect accuracy in the understanding of treatment risk reduction is patients' proficiency in the language in which risk information is communicated. Immigrant populations can have limitations in nonnative language proficiency. Therefore, when risk information is not provided in the native language of patients from such populations, the detrimental effect of denominator neglect on estimates of treatment risk reduction can be amplified. This is highly relevant to modern societies, which are increasingly becoming culturally heterogeneous [14, 48]. Furthermore, it has been observed that immigrants with limited nonnative language proficiency are in many cases at the greatest risk of illness [49, 50]. In sum, immigrant groups with low-risk literacy or limited nonnative language proficiency can have a reduced access and understanding of medical risks [51–53], thus mitigating the effectiveness of public health strategies [54–56].

To what extent do limitations in nonnative language proficiency affect understanding of treatment risk reduction in immigrant populations? This question was addressed by Garcia-Retamero and Dhami [30] in a study involving participants who were all Polish immigrants to the United Kingdom (*n* = 96). As in the study by Garcia-Retamero and Galesic [31], the authors investigated participants' tendency to show denominator neglect when judging the effectiveness of treatments using information from unequally sized groups of treated and nontreated patients (i.e., inconsistent denominators). Additionally, Garcia-Retamero and Dhami [30] investigated the extent to which icon arrays could help to reduce denominator neglect when risk information was not provided in participants' native language.

A mixed design with three independent variables was employed in the study. First, the sizes of the denominators were manipulated within subjects and had four levels (see Table 1). Second, the provision of icon arrays was manipulated between subjects and had two levels: icons in addition to the numerical information about risk reduction (see Figure 1), and no icon arrays (i.e., numerical information only). Finally, language was a between-subjects factor and had two levels: information about treatment risk reduction was provided either in participants' native language, Polish, or in a nonnative language, English. Participants' estimates of treatment risk reduction were measured following the procedure used by Schwartz et al. [10].

**Figure 3:**
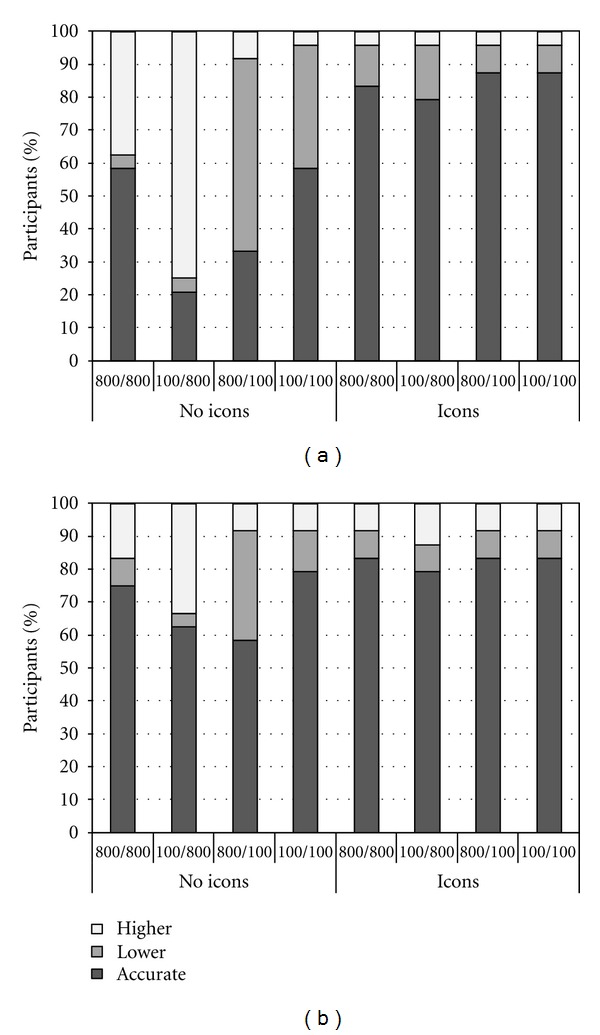
(a) Percentage of participants whose estimates of risk reduction were accurate, lower, or higher than the exact value as a function of the sizes of the denominators and icon arrays when information about risk reduction was provided in *English*. (b) Percentage of participants whose estimates of risk reduction were accurate, lower, or higher than the exact value as a function of the sizes of the denominators and icon arrays when information about risk reduction was provided in *Polish*.

Results in this study were consistent with those reviewed above (see Figures 3(a) and 3(b)). When information about the drug was provided numerically and the sizes of the denominators were different, many participants provided inaccurate estimates of treatment risk reduction. Again, a tendency to focus on absolute numbers in numerators instead of taking proportions into account (i.e., denominator neglect) can account for these patterns of inaccurate estimates. Importantly, this tendency was particularly pronounced when the information was provided in English rather than in Polish. Furthermore, when the sizes of the denominators were equal or when they were different and icon arrays were added to the numerical information, denominator neglect was significantly reduced. This increase in accuracy was more prominent when information about treatment risk reduction was not provided in participants' native language, presumably because they discarded the verbal description of the numerical information and focused solely on information in the icon arrays.

## 4. The Impact of Graph Literacy on the Assessment of Treatment Risk Reduction

 As the studies reviewed above suggest, visual displays such as icon arrays can significantly improve understanding of ratio concepts. However, graphs are not equally useful for all individuals [26, 39, 57]. Recent research has shown that people differ substantially in their ability to understand graphically presented information, or *graph literacy* [32, 58]. Individuals with high graph literacy have been found to make more elaborate inferences when viewing graphical displays, as compared with less graph-literate individuals. For instance, highly graph-literate individuals extract information of a higher level of complexity when viewing line graphs [59] and they are more capable of making main effect inferences for bar graphs than individuals with low graph literacy [60].

To what extent can individual differences in graph literacy affect understanding of treatment risk reduction when this information is presented visually? Okan et al. [32] addressed this question in an experiment in which individuals with varying levels of graph literacy evaluated treatment risk reduction using information from unequally sized groups of treated and nontreated patients in numerical and visual formats. The rationale of this study was analogous to that of the studies reviewed above. Additionally, graph literacy was measured using an instrument developed by Galesic and Garcia-Retamero [58]. The instrument consists of 13 items and measures both basic graph-reading skills and more advanced comprehension for different types of graphs—including line plots, bar charts, pies, and icon arrays. The psychometric properties of the instrument have been assessed in a survey conducted on probabilistically representative samples of people from Germany and the United States (see Galesic and Garcia-Retamero [58]). Okan et al. [32] split participants (*n* = 168) into two groups according to the median graph literacy score for the total sample (i.e., 10).

 Two independent variables were manipulated in this study. First, the sizes of the denominators were manipulated within subjects and had four levels. As in the previous studies, denominators were set to be 800/800, 100/800, 800/100, or 100/100. However, in this case, numerators were adjusted in such a way that relative risk reduction was always 80% (see Table 2). Second, as in previous studies the presentation of icon arrays was manipulated between subjects by providing half of the participants with icon arrays, in addition to the numerical information. Estimates of treatment risk reduction were measured following the procedure used by Schwartz et al. [10] described above.

In line with the previously reviewed studies, when information about the drug was provided numerically and the sizes of the denominators were different, many participants provided inaccurate estimates. Icon arrays helped people to take into account both the overall number of treated and nontreated patients in their estimations of treatment risk reduction. Namely, when the sizes of denominators were different and icon arrays were presented alongside numerical information, the percentage of correct estimates increased from 42% to 73%, and from 34% to 81% for the 100/800 and 800/100 conditions, respectively.

**Figure 4:**
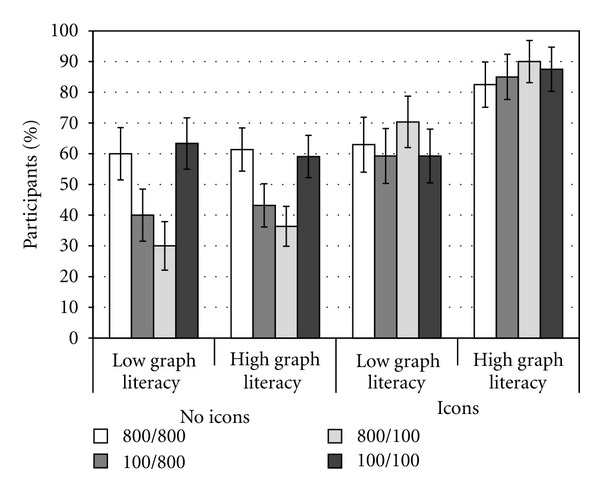
Percentage of participants whose estimates of risk reduction were accurate, as a function of graph literacy, icon arrays, and sizes of the denominators. Error bars represent one standard error.

Crucially, graph literacy was found to moderate the effectiveness of icon arrays. When icon arrays were not provided, 48% of the participants with low graph literacy provided correct estimates, compared with 64% when icon arrays were provided. For participants with high graph literacy, the increase in the percentage of correct estimates was significantly larger, rising from 51% to 87% (see Figure 4). Over all, these findings call attention to the notion that the usefulness of visual aid can in some cases be mitigated by the lack of viewers' graph-related knowledge.

## 5. Discussion and Conclusions

Understanding numerical information is essential for informed decision making [61]. Unfortunately, numerical information can be presented in ways that bias and undermine accurate judgment and decision making. A prominent example is *denominator neglect*, or the focus on the number of times a target event has happened, without consideration of the overall number of opportunities for it to happen. The studies reviewed here demonstrate the existence of a robust tendency for people to show denominator neglect, disregarding the overall number of treated and nontreated patients in favor of the number of treated and nontreated patients who died. These findings are in line with evidence from Epstein and colleagues in lottery gambles [20, 62–64] and with research by Chapman [65] (see also [66, 67]), who showed that problems in which a denominator is shared (one-sample problems) or equal (two-sample equal sample size problems) are easier to solve than problems in which denominators differ across options. Finally, as noted above, Yamagishi [22] has similarly shown that causes of death with greater absolute numbers are perceived as more risky even if they have smaller proportions than others with smaller absolute numbers. 

The studies reviewed in the present paper demonstrate that denominator neglect is more prominent both among individuals with low numeracy when information about treatment risk reduction is expressed numerically, and in those with limited nonnative language proficiency when this information is not expressed in their native language. That is, individual differences in skills such as numeracy or language proficiency tend to affect the likelihood of judgment errors that can have important consequences for decisions about health. These findings indicate that patients with low numeracy and ethnic minorities with limited nonnative language proficiency will be at greater risk of illness (see also [49, 50, 55]). Epidemiologic research has long shown that these populations suffer disproportionately from several diseases [35, 36, 68]. Immigrant groups also differ from the indigenous population in their reports of pain, the way they communicate symptoms, their beliefs about the cause of illness, and their understanding of concepts such as “risk factors” or “being at risk” [51, 52, 69–71].

Similarly, patients with low numeracy have less accurate perceptions of the risks and benefits of screening [10, 72–74] and are more susceptible to errors in judgments and decisions than those with high numeracy [16, 75–77], which reduces their medication compliance and impairs risk communication [17]. These patients are also especially vulnerable to adherence problems when following a dosing regimen [35], and have a longer history of hospitalization [36]. Finally, patients with low numeracy tend to be more susceptible to being influenced by the way the health information is framed [11, 37], and have more difficulty accurately recalling numerical information about health [75]. The findings reviewed here add to this literature showing that patients with low numeracy and limited language skills also tend to disregard crucial information when assessing treatment risk reduction. The current review also suggests that one likely explanation is that pertinent health messages do not reach these groups effectively. For immigrant populations, translated resources can offer a promising approach to communicating health information to immigrants but may not always be sufficient [54, 78, 79].

 The finding that people—especially those with low numeracy skills and limited nonnative language proficiency—tend to disregard crucial information when making important decisions about their health is a troubling finding with public health implications. Fortunately, the studies reviewed here converge to point to a potentially effective method for overcoming denominator neglect: Providing icon arrays in addition to numerical information helps people make more accurate assessments of risk reduction. Nevertheless, it should be noted that people with low graph literacy benefit to a lesser extent from these visual displays [32, 39]. Thus, individuals with low graph literacy may require especially designed formats such as analogies (e.g., [80]) and/or additional training in the use of graphs.

The results outlined in the current review support and extend previous research indicating that visual aids often facilitate risk communication in the health domain [7, 28, 37, 39, 45, 81, 82]. In particular, they support the hypothesis put forward by Stone et al. [82] (see also Ancker et al., [26]), stating that graphical formats displaying both foreground information (e.g., number of people harmed) and background information (e.g., number of people at risk) can contribute to focus people's attention on the background too, bringing attention to the relationship between the numerator and the denominator (see also Lipkus [57]). Additionally, these results extend the literature on denominator neglect as they provide support for Reyna and Brainerd's [16] hypothesis that visual displays can help people represent superordinate classes (i.e., the overall number of patients who did and did not receive a treatment), thus allowing people to disentangle classes that are overlapping in ratios. Of note, the findings reviewed also indicate that individuals with low graph literacy may find it difficult to associate the visual patterns contained in icon arrays with meaningful interpretations of the data represented [32].

The studies reviewed also have implications for medical practice as they suggest suitable ways to communicate complex quantitative medical data to people who are disadvantaged by their lack of numerical and/or language skills—people who may also be struggling to cope with fear and uncertainty associated with major illnesses or medical interventions. At the policy level, the current review accords with a medical convention of reporting risks using ratios that have the same denominator [27]. However, patients not only receive health-related information from their physicians but they also often obtain this information from a number of other sources such as the media, the Internet, and their friends and relatives [83, 84]. These alternative sources often do not use the most effective formats for presenting the health information [85, 86]. When the common practice of communicating risks using ratios with the same denominator is not feasible, adding visual displays to information about risks should be an effective method for enhancing comprehension in populations disadvantaged by limited numerical skills or language proficiency. In contrast, if the goal is to persuade patients rather than enhance their informed decision making (e.g., cessation of smoking), using ratios with different denominators may be most effective. This seemingly exploitative approach may be considered justifiable in situations aiming to achieve health gain—however, given the power to distort and induce errors in judgment any such use should be subject to bioethics review and approval.

A number of open questions remain to be addressed in future research. For instance, it would be interesting to achieve a precise specification of the relations between the set of individual differences outlined above and the cognitive processes that mediate the differences. Individual differences that can influence risky judgment and decision making include decision making styles [87–90], specific expertise [91, 92], and domain general cognitive abilities [21, 93, 94]. Research indicates that general decision making skills have significant relations among them and with other measures of cognitive abilities and styles [95, 96]. Additionally, the studies reviewed emphasize the importance of considering the fit between (i) persons, (ii) cognitive processes, and (iii) task environments when designing interventions such as visual aids. Future work should directly aim to trace attentional and cognitive processes underlying the effect of visual aids, including icon arrays and also other kinds of displays such as bar charts or line plots. This is an essential step in efforts to facilitate the development of psychologically sensitive training methods that enhance the understanding of quantitative medical information for disadvantaged individuals. Ultimately, the studies outlined above emphasize the importance and value of working towards the development of custom-tailored risk communication interventions that are sensitive to the various needs and abilities of diverse individuals who must make potentially life changing decisions.

## Figures and Tables

**Table 1:** Number of treated and nontreated patients who died from a heart attack used in fictitious medical scenarios.

Sizes of denominators^a^	Treated patients		Nontreated patients	
	Dead patients	Population size	Dead patients	Population size
800/800	40	800	80	800
100/800	5	100	80	800
800/100	40	800	10	100
100/100	5	100	10	100

Note. Treatment risk reduction is 50% in all conditions.

^a^Treated and untreated patients, respectively.

**Table 2:** Number of treated and nontreated patients who died from a heart attack used in fictitious medical scenarios.

Sizes of denominators^a^	Treated patients		Nontreated patients	
	Dead patients	Population size	Dead patients	Population size
800/800	16	800	80	800
100/800	2	100	80	800
800/100	16	800	10	100
100/100	2	100	10	100

Note. Treatment risk reduction is 80% in all conditions.

^a^Treated and untreated patients, respectively.
